# Speech Perception and Dichotic Listening Are Associated With Hearing Thresholds and Cognition, Respectively, in Unaided Presbycusis

**DOI:** 10.3389/fnagi.2022.786330

**Published:** 2022-02-24

**Authors:** Mariela C. Torrente, Rodrigo Vergara, Felipe N. Moreno-Gómez, Alexis Leiva, Simón San Martin, Chama Belkhiria, Bruno Marcenaro, Carolina Delgado, Paul H. Delano

**Affiliations:** ^1^Departamento Otorrinolaringología, Facultad de Medicina, Universidad de Chile, Hospital Clínico Universidad de Chile, Santiago, Chile; ^2^Departamento de Kinesiología, Facultad de Artes y Educación Física, Universidad Metropolitana de Ciencias de la Educación, Santiago, Chile; ^3^Centro Nacional de Inteligencia Artificial CENIA, Santiago, Chile; ^4^Departamento de Biología y Química, Facultad de Ciencias Básicas, Universidad Católica del Maule, Talca, Chile; ^5^Departamento de Neurociencia, Facultad de Medicina, Universidad de Chile, Santiago, Chile; ^6^Facultad de Medicina, Biomedical Neuroscience Institute (BNI), Universidad de Chile, Santiago, Chile; ^7^Departamento de Neurología y Neurocirugía, Hospital Clínico de la Universidad de Chile, Santiago, Chile; ^8^Centro Avanzado de Ingeniería Eléctrica y Electrónica, AC3E, Universidad Técnica Federico Santa María, Valparaíso, Chile

**Keywords:** presbycusis, age-related hearing loss, auditory processing, cognition, elderly, hearing aids

## Abstract

Presbycusis or age-related hearing loss is a prevalent condition in the elderly population, which affects oral communication, especially in background noise, and has been associated with social isolation, depression, and cognitive decline. However, the mechanisms that relate hearing loss with cognition are complex and still elusive. Importantly, recent studies show that the use of hearing aids in presbycusis, which is its standard management, can induce neuroplasticity and modify performance in cognitive tests. As the majority of the previous studies on audition and cognition obtained their results from a mixed sample of subjects, including presbycusis individuals fitted and not fitted with hearing aids, here, we revisited the associations between hearing loss and cognition in a controlled sample of unaided presbycusis. We performed a cross-sectional study in 116 non-demented Chilean volunteers aged ≥65 years from the Auditory and Dementia study cohort. Specifically, we explored associations between bilateral sensorineural hearing loss, suprathreshold auditory brain stem responses, auditory processing (AP), and cognition with a comprehensive neuropsychological examination. The AP assessment included speech perception in noise (SIN), dichotic listening (dichotic digits and staggered spondaic words), and temporal processing [frequency pattern (FP) and gap-in-noise detection]. The neuropsychological evaluations included attention, memory, language, processing speed, executive function, and visuospatial abilities. We performed an exploratory factor analysis that yielded four composite factors, namely, hearing loss, auditory nerve, midbrain, and cognition. These four factors were used for generalized multiple linear regression models. We found significant models showing that hearing loss is associated with bilateral SIN performance, while dichotic listening was associated with cognition. We concluded that the comprehension of the auditory message in unaided presbycusis is a complex process that relies on audition and cognition. In unaided presbycusis with mild hearing loss (<40 dB HL), speech perception of monosyllabic words in background noise is associated with hearing levels, while cognition is associated with dichotic listening and FP.

## Introduction

Age-related hearing loss (ARHL), or presbycusis, affects one of every three persons aged more than 65 years, with an estimated worldwide prevalence of ∼430 million people (World Health Organisation [WHO], 2021). Presbycusis is produced by neurodegenerative processes of peripheral and central auditory structures (Gates and Mills, 2005), which, at the clinical level, is characterized by bilateral high-frequency hearing loss and deteriorated speech intelligibility (Van Eyken et al., 2007). Oral communication relies on a series of neural mechanisms involving hearing and cognitive functions (Ruggles et al., 2011; Pienkowski, 2017). As these functions deteriorate with aging (Quaranta et al., 2014; Atcherson et al., 2015), individuals develop communication deficits that can be attributed to presbycusis and cognitive decline. Moreover, ARHL has been associated with social isolation and depression, and it has been recognized as a modifiable risk factor for dementia (Livingston et al., 2017, 2020). However, the mechanisms that relate to hearing loss and cognitive decline are complex and still under research (Yue et al., 2021).

There are several publications that explore the relationship between cognition and auditory processes in aged populations, with conflicting results. For example, Anderson et al. (2013) described a negative association between speech discrimination in noise (SIN) and cognition, while Murphy et al. (2018) found no correlation between SIN and working memory. The cognitive load has been proposed as an important factor to explain discrepancies between studies exploring auditory and cognitive functions. For instance, Nixon et al. (2019) found no association between free recall of two digits and cognition, while Fischer et al. (2017) described a significant association between cognition and free recall of three digits, showing that a harder cognitive challenge could explain the significant associations between audition and cognition.

Another important variable that could have a role in modifying the interactions between cognitive and auditory functions is the neuroplasticity derived from the use of hearing aids in subjects with hearing loss. In this line, recent reports show that the use of hearing aid devices can induce neuroplasticity and modify the performance in cognitive tests (Glick and Sharma, 2020; Vogelzang et al., 2021). These recent findings should be considered as an important caveat for future studies, due to the fact that the majority of previous studies on audition and cognition used mixed data from subjects with and without the use of hearing aids (O’Brien et al., 2021), or do not report whether individuals were aided or not with auditory devices such as hearing aids or cochlear implants (Humes et al., 1994, 2013; Sheft et al., 2015; Murphy et al., 2018).

In this study, we proposed that to better understand the interactions between cognition and hearing functions, these should be studied in a controlled group of individuals, without the influence of neuroplasticity induced by hearing aids. Therefore, in this study, we aimed to examine the associations between auditory and cognitive functions in non-demented subjects (≥65 years) without previous use of hearing aids. We studied audiogram hearing thresholds; suprathreshold auditory brain stem responses (ABR); speech perception in noise (SIN); dichotic listening [dichotic digits and staggered spondaic words (SSW)]; temporal processing [frequency pattern (FP) and gap-in-noise (GIN) detection]; and cognitive skills including attention, memory, language, processing speed, executive function, and visuospatial abilities.

## Materials and Methods

### Subjects

A total of 134 volunteers (≥65 years), with a Mini-Mental State Examination (MMSE) score > 24, were prospectively involved (between 2016 and 2018) in the Auditory and Dementia study (ANDES) cohort. They were all Chileans, belonging to the Recoleta and Independencia districts from Santiago, Chile, and spoke Spanish as their native language. Thirteen subjects did not complete the audiological evaluation, and two patients had missing data. In addition, following the recommendations of the American Academy of Audiology (2010) guidelines for the evaluation of auditory processing (AP), three subjects that had the best ear pure-tone average (PTA) greater than 50 dB HL were not considered for AP evaluations. The exclusion criteria were previous ear disease, previous use of hearing aids, asymmetrical hearing loss (defined as a difference greater than 15 dB HL in at least two contiguous frequencies), conductive hearing loss (defined as a PTA air-bone gap greater than 10 dB HL), stroke, dementia, and major psychiatric and neurological disorders. All volunteers gave written informed consent in accordance with the Declaration of Helsinki. All procedures were approved by the Ethics Committee of the Clinical Hospital of the University of Chile, permission number: OAIC752/15.

### Hearing Assessment

#### Pure-Tone Audiogram Thresholds

We recorded air and bone conduction thresholds for octaves between 125 and 8,000 Hz for all subjects with an AC40 audiometer (Interacoustics™^®^, Middelfart, Denmark), DD45 headphones, and B-71 bone oscillator, according to the clinical standards of ANSI S3.6, 2010. The average hearing threshold for frequencies 0.5, 1, 2, and 4 kHz (PTA) was calculated and used for subsequent analysis.

#### Distortion-Product Otoacoustic Emissions

In a previous study, we associated the loss of distortion-product otoacoustic emissions (DPOAE) with cingulate cortex atrophy (Belkhiria et al., 2019), showing that the presence of DPOAE is an important factor to include for modeling the relationship between auditory and cognitive functions in elderly people. DPOAE were measured using an ER10C microphone with built-in sound sources (Etymotic Research, Elk Grove Village, United States), presenting eight pairs of primary tones (f1 and f2, at 65 and 55 dB SPL, f2/f1 ratio of 1.22) in each ear at eight different 2f1–f2 frequencies: 707, 891, 1,122, 1,414, 1,781, 2,244, 2,828, and 3,563 Hz. To consider the presence of a DPOAE, we used an amplitude criterion: the amplitude of a given DPOAE (dB SPL) should be at least 6 dB above the noise floor (Belkhiria et al., 2019). Using this criterion, we counted the number of detectable DPOAEs per ear, going from 0 to 8, where “0” meant that the subject had no detectable DPOAE in that ear and “8” meant that the subject had detectable DPOAEs at all tested frequencies in that ear. In contrast to a method that only measured the amplitude of DPOAEs (SNR, signal-to-noise ratio), the procedure of counting the number of detectable DPOAEs per ear allowed us to evaluate the cochlear function in the entire sample, without eliminating subjects with no detectable DPOAEs (and no measurable amplitude in dB SPL), which is frequent in elderly people.

#### Suprathreshold Auditory Brainstem Responses

Previously, we showed that the suprathreshold amplitude of ABR responses is associated with the thickness of temporal and parietal cortices (Delano et al., 2020). For this reason, in this study, we included the measurement of suprathreshold ABR waves I and V. We used an Eclipse EP25 with research licensed equipment (Interacoustics™^®^, Middelfart, Denmark) to elicit ABR. The stimuli were broadband clicks delivered through E-A-RTONE™ 3A inserts earphones, with an intensity of 80 dB nHL, and a duration of 100 μs. We used high pass 100 Hz filters and low pass 3,000 Hz filters. Responses were recorded using active electrodes placed on both mastoids and on the forehead (reference or non-inverting), and a ground electrode was secured over the right brow. Waves I to V were identified from two averages of 2,000 repetitions. The amplitudes of waves I and V were defined from the peaks of the respective waves and the negative troughs that followed, and latency from peaks. Amplitude and latency of wave V were measurable in all subjects, while wave I was identified in 109/116 (93.9%) of the cases. When waves I were missing (with detectable wave V), they were imputed with the lower observed value for the amplitude of wave I and the greater observed value for the latency of wave I (Delano et al., 2020).

#### Auditory Processing Evaluation

The battery chosen for the evaluation of AP was developed considering the recommendations of the American Speech-Language-Hearing Association [ASHA], 2005) and the American Academy of Audiology (2010). Speech tests were available in Spanish and were previously validated in Chile. Speech and non-speech tests were selected from the following categories: dichotic speech, monoaural low-redundancy speech tests, and temporal processing. All tests, except the GIN test, were performed with a commercially available recording system (Auditec™^®^, St. Louis, MO, United States), delivered to the participant through the AC40 audiometer and DD45 headphones. Before testing each person, we calibrated the audiometer output following the instructions of the manufacturer using a 1 kHz pure tone. According to the availability of evaluations in Spanish, we included the following tests.

##### Speech in Noise

Lists of 25 monosyllabic words were presented monaurally to each ear with a white noise background at a 10 dB SNR. The result for each ear was the total number of correct answers expressed as a percentage. The sound level presentation was 40 dB above audiogram thresholds (Fuente and McPherson, 2006).

##### Dichotic Digits

Following a binaural presentation of 20 sequences of digit pairs, subjects had to repeat the four-number sequence (two pairs for each ear, free recall). The result for each ear was the total number of correct repetitions for the digits presented to each ear expressed as a percentage. The sound level presentation was 50 dB above PTA (Fuente and McPherson, 2006).

##### Spanish Version of Staggered Spondaic Words Test

Participants were exposed to 40 sequences of four words binaurally. The result for each ear was the sum of errors for the competing and non-competing performance, that is, the total number of errors for the right and left ears. The level of presentation was 50 dB above PTA (Cañete et al., 2020). To minimize peripheral interference, the results were corrected by the word discrimination score, and subjects with best ear PTA over 50 dB HL were excluded (Arnst and Doyle, 1983).

##### Gap Detection Threshold

We used the beta adult version of the Adaptive Test of Temporal Resolution©, with the across channel modality (Lister et al., 2011). Briefly, the subject was exposed to a stimulus that included a silent gap of adaptive duration between two bands of narrowband noise: the first centered in 1.1 kHz and the second in 2 kHz. The results reflected the smallest gap duration in milliseconds (ms) that the patient could detect. The level of presentation was the maximum intensity tolerated by the patient, which was between 50 and 70 dB above PTA in most cases, in the right ear only. Nine volunteers were not able to execute the GIN test (8%) and were subsequently excluded from the analysis of GIN performance.

##### Frequency Pattern

Three tones were presented monaurally and randomly in a set of 30 sequences, with either high (1,122 Hz) or low pitch (880 Hz) (Sanchez et al., 2008). Participants had to identify the correct three-tone sequences of the low- and high-pitch stimuli; for example, high-high-low or low-low-high. The results refer to correct answers per ear. The level of presentation was 50 dB above PTA.

### Cognitive Evaluation

Cognitive performance was assessed by an experienced psychologist in cognitive tests that were blind to auditory evaluations. Instructions were given orally and visually using a desktop computer. The battery included the Trail Making Test Part A (TMT-A) for processing speed and Part B (TMT-B) for executive functions (Arango-Lasprilla et al., 2015), Wechsler Digit Symbol for processing speed (Peña-Casanova et al., 2009), the Boston Naming Test for language in an abbreviated version of 30 items (Kaplan et al., 1978), the Rey-Osterrieth Complex Figure Test for visuospatial abilities (Rey, 1959), the Forward and Backward Digit Span for verbal working memory and attention (Peña-Casanova et al., 2009), and the total recall of the Free and Cued Selective Reminding Test (FCSRT) to explore verbal episodic memory (Grober et al., 1988).

### Statistical Analysis

For descriptive analysis, we used the median and interquartile range (IQR) of demographic variables. We also explored gender differences using the Mann-Whitney *U* test and ear differences for auditory processes using the Wilcoxon rank-sum test.

We performed exploratory factor analyses for hearing loss, auditory brain stem, and cognitive domains, allowing us to build composite scores that represented the main aspects of the auditory processes analyzed. To define how many scores were produced by each of these elements, we used the parallel analysis method (Hayton et al., 2004). Factors were extracted with principal axis factorization rotated using oblimin (Costello and Osborne, 2005), and each factor was submitted to Cronbach’s alpha internal consistency analysis (Cronbach, 1951) using only the variables with loadings greater than 0.3. Composite scores were estimated using the regression method based on exploratory factor analyses results. The following variables were used for each factor analysis:

• Hearing loss: PTA and DPOAE of both ears.
• Suprathreshold ABR: latency and amplitude of waves I and V of both ears.
• Cognitive domains: forward and backward digit span, digit symbol, TMT-A, TMT-B, Rey figure, total recall of the FCSRT, and Boston naming test.

Once hearing loss, suprathreshold ABR, and cognitive domain scores were defined and estimated, we evaluated the differential contribution of these elements on auditory processes using generalized multiple linear regression models. Each category of AP was explored independently and not combined in a composite score. We chose this theoretical-driven approach rather than a data-driven approach to avoid artifactual results (Costello and Osborne, 2005). As dependent variables, we included the performance of each ear in SIN, dichotic digits, SSW, and FP tests. All our dependent variables, except for the GIN task, were performance percentages with bounded scores from individual answers, which could be right or wrong. These kinds of variables are better modeled using a binomial modeling approach (Crawley, 2013). In the case of obtaining overdispersion, we used a quasibinomial approach. In the case of the GIN task, variables that respond to waiting time events, and are zero bounded, are expected to have gamma distributions (Nobel and Tijms, 2006). For this reason, we modeled the GIN task assuming gamma distribution. As independent variables, we included the composite scores derived from the exploratory factor analyses (EFA), as well as sex, years of education, and age. Since many of these evaluations required not only a proper cochlear function but also comprehension of the task, we included an interaction between cognition and hearing loss factors. Non-significant regressors (including interaction) were removed from the models using a backward method followed by a forward method. Both results and procedures were manually compared. When the solution was not convergent, we kept the solution presenting the highest pseudo-*R*-squared value. To estimate pseudo-*R*-squared values, we used a variance function-based method (Zhang, 2020). The *p*-values were corrected using Bonferroni’s method. A model was significant if the pseudo-*R*-squared values were bigger than 0.15 (Schober and Schwarte, 2018). All statistical analyses were performed with the *R* project, with an alpha value of 0.05.

## Results

We included 116 volunteers, with a median age of 73 years (IQR: 8 years), median education of 11 years (IQR: 6 years), and a median hearing threshold of 27.5 dB HL (IQR: 17.2 dB HL) for the right ear and 26.3 dB HL (IQR: 21.3 dB HL) for the left ear. Women (62% of the sample) were younger, had better hearing (both PTA and DPOAE), had larger amplitude and shorter latency of wave I of the left ear, shorter latency of wave V for both ears, performed better in SSW for both ears and FP of the right ear, and performed worse in digit symbol and total recall tests (Table 1). Due to these differences, the following analysis included gender as a variable.

**TABLE 1:** Description (mean and SD) of variables, including demographics (age and schooling), hearing loss, suprathreshold auditory brain stem responses, auditory processes, and cognitive domains.

Variable	Median (IQR)		
	Men, *n* = 44 (38%)	Women, *n* = 72 (62%)	Mann-Whitney *U*, *p* value (two-tailed)
Age (years)	75 (8)	72 (8)	0.012
Schooling (years)	11 (6)	11 (6)	
PTA right ear (dB HL)	34.4 (18.1)	25 (16.7)	0.042
PTA left ear (dB HL)	32.5 (22.5)	23.8 (18.2)	0.005
OAE right ear	1.0 (4)	4.0 (7)	0.004
OAE left ear	1.5 (5)	5.0 (7)	0.026
Amplitude wave I right ear (μV)	0.1 (0.09)	0.12 (0.1)	
Amplitude wave I left ear (μV)	0.10 (0.13)	0.14 (0.11)	0.012
Latency wave I right ear (ms)	1.53 (0.26)	1.53 (0.17)	
Latency wave I left ear (ms)	1.53 (0.34)	1.50 (0.19)	0.024
Amplitude wave V right ear (μV)	0.33 (0.21)	0.38 (0.17)	
Amplitude wave V left ear (μV)	0.36 (0.16)	0.36 (0.19)	
Latency wave V right ear (ms)	5.73 (0.33)	5.60 (0.37)	0.000
Latency wave V left ear (ms)	5.73 (0.47)	5.60 (0.3)	0.001
Speech in noise right ear (correct answers)	22 (2)	23 (2)	0.042
Speech in noise left ear (correct answers)	22 (3)	23 (2)	
Dichotic digits right ear (correct answers)	37 (5)	35 (4)	
Dichotic digits left ear (correct answers)	29 (13)	29 (10)	
SSW right ear (total of errors)	3 (4)	2 (3)	0.032
SSW left ear (total of errors)	8 (8)	4 (7)	0.001
Frequency pattern right ear (correct answers)	13 (9)	11 (10)	0.031
Frequency pattern left ear (correct answers)	11 (10)	9 (12)	
Gap in noise (ms)	87.1 (67.7)	99.7 (97.9)	
Rey figure	31.5 (4.5)	31.0 (5)	
Foward digit span	6 (3)	6 (2)	
Backward digit span	4 (1)	4 (2)	
Digit symbol	34.5 (17)	40.0 (23)	0.012
TMT-A	57.5 (32)	49.0 (34)	
TMT-B	200 (194)	140 (151)	
FCSRT	43 (8)	46 (4)	0.001
Boston nominating test	25 (4)	26 (5)	

*Differences by gender were analyzed for each variable.*

*PTA, pure-tone average of responses for 0.5, 1, 2, and 4 kHz; OAE, total number of distortion product otoacoustic emissions; SSW, staggered spondaic words; TMT-A, Trail Making Test Part A; TMT-B, Trail Making Test Part B; FCSRT, total recall of the Free and Cued Selective Reminding Test; IQR, interquartile range.*

**FIGURE 1:**
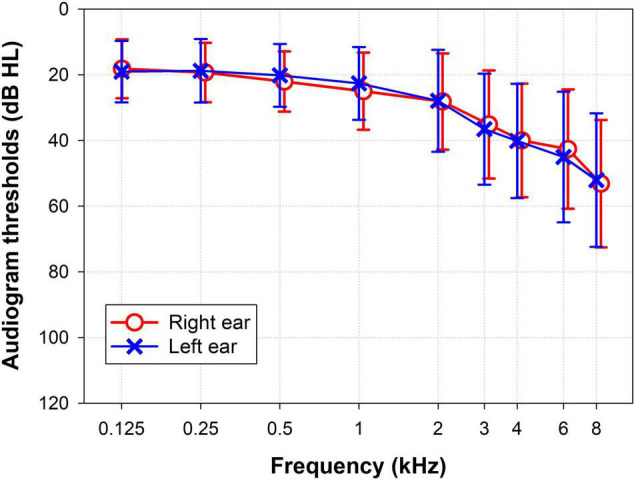
Grand average audiogram for pure-tone thresholds expressed as mean and SD of the left (blue crosses) and right (red circles) ears.

The majority of the subjects had either normal hearing, defined as PTA less than 25 dB HL (49.1%), or mild hearing loss, defined as PTA between 25 and 40 dB HL (36.2%). Only 14.7% of subjects had PTA greater than 40 dB HL. The audiometric profile for both ears was a descending sensorineural hearing loss (Figure 1). The left ear had better hearing levels and larger wave I amplitude (Table 2). The results of AP tests showed significant differences between ears for dichotic listening (dichotic digits and SSW) and for FP (Table 2). Further analyses were dichotomized by ear due to these differences.

**TABLE 2:** Performance of both ears in tests of hearing and auditory processing.

	Right ear (median ± SD)	Left ear (median ± SD)	Wilcoxon signed rank test *p* value
PTA	28.85 ± 11.23	27.83 ± 11.57	**0.007**
DPOAE	3.31 ± 2.87	3.34 ± 3.0	0.97
Amplitude wave I	0.11 ± 0.7	0.13 ± 0.08	**0.03**
Amplitude wave V	0.37 ± 0.13	0.4 ± 0.15	0.06
Latency wave I	1.59 ± 0.21	1.58 ± 0.22	0.43
Latency wave V	5.65 ± 0.25	5.66 ± 0.25	0.89
Speech in noise	23 ± 2.6	23 ± 2.4	0.127
Dichotic digits	36 ± 4.7	29 ± 7.8	**<0.000**
Staggered spondaic words (errors)	2 ± 5.9	6 ± 11	**<0.000**
Frequency pattern	10 ± 7.6	9 ± 7.5	**0.013**

*The left ear had a better hearing level (PTA) and larger amplitude of wave I in suprathreshold ABR. There was a significant difference in favor of the right ear for binaural integration (dichotic digits and staggered sporadic words) and frequency pattern. Speech in noise: correct answers out of 25. Dichotic digits: correct answers out of 40.*

*Staggered spondaic words: total number of errors considering both competing and non-competing. Frequency pattern: correct answers out of 30.*

*PTA: pure-tone threshold average for frequencies 0.5, 1, 2, and 4 kHz; DPOAE, distortion product otoacoustic emissions.*

*Bold numbers are used to highlight statistically significant values.*

The EFA yielded four composite scores. One factor for hearing loss (DPAOE and PTA) with a Cronbach’s alpha of 0.94. Suprathreshold ABR EFA produced two factors: amplitude and latency of wave I of both ears (Cronbach’s alpha: 0.72) and amplitude and latency of wave V of both ears (Cronbach’s alpha: 0.71). These were nominated waves I (auditory nerve) and V (midbrain), respectively. Cognitive EFA had only one factor, Cronbach’s alpha of 0.79.

**FIGURE 2:**
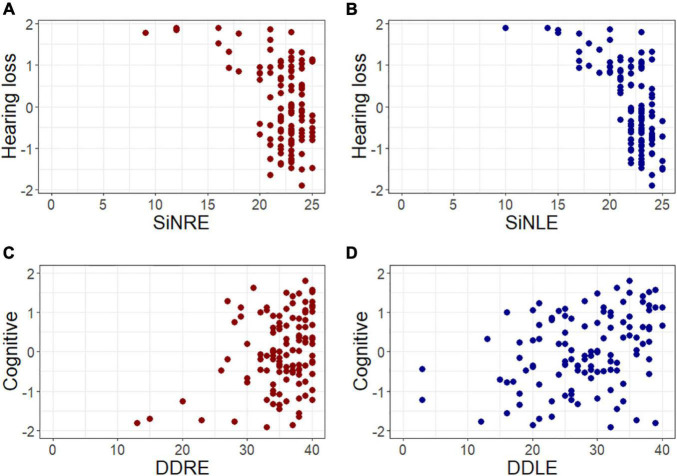
Speech perception and dichotic listening are associated with hearing thresholds and cognition, respectively. Scatter plots presenting relevant associations found using generalized linear models. The panels present the association between **(A)** hearing loss and speech in noise for the right ear (SINRE), **(B)** hearing loss and speech in noise for the left ear (SINLE), **(C)** cognitive score and dichotic digits of the right ear (DDRE), and **(D)** cognitive score and dichotic digits of the left ear (DDLE). All variables are presented in *z*-score, red circles represent right ear evaluations, while blue circles illustrate left ear assessments.

Next, we fitted models including the variables age, sex, and schooling, and the four factors found with EFA (hearing loss, auditory nerve, midbrain, and cognition) as independent variables for each of the auditory processes evaluated, included as dependent variables (Table 3). Hearing loss was a significant regressor in the SIN model, consistently presenting the highest standardized regression coefficients. Figures 2A,B shows the relation between the hearing loss factor and SIN of the right and left ears, illustrating that a greater hearing loss is associated with bilateral poor SIN performance. In addition, the model of SIN included age and wave I for the right ear as significant regressors. Regarding dichotic listening, SSW and DD showed significant associations with cognition. In the case of SSW, hearing loss and cognition explained 27% of the variance of SSW for the left ear, and 17% of the variance for the right ear, while DD was only significantly associated with the cognition factor, explaining 18 and 12% of the variance (right and left ear correspondingly, Table 3). Figures 2C,D shows the relationship between the cognitive factor and DD of the right and left ears, illustrating that a better bilateral performance in the DD tests is associated with a better cognitive performance. For FP, the variable of cognition explained 31 and 24% of the variance (right ear and left ear, respectively), while wave V was also a significant variable but only for the left ear. The other models were not further commented given their low pseudo-*R*-squared values (<0.15).

**TABLE 3:** Generalized multiple linear regression models for performance in auditory processes (SIN, speech in noise; DD, dichotic digits; SSW, staggered spondaic words; FP, frequency pattern; GiN, gap in noise) for both ears (RE, right ear; LE, left ear).

	SINRE	SINLE	DDRE	DDLE	SSWRE	SSWLE	FPRE	FPLE	GIN
Hearing	−0.38***	−0.51***			0.51***	0.48***			
Auditory nerve	−0.17*								
Midbrain								0.28**	
Cognition		0,06	0.30***	0.34***	−0.30*	−0.36***	0.64***	0.57***	0.001**
Schooling									
Age	−0.17*	−0.13*							
Gender (M)							0.61**		
Pseudo *R*^2^	**0.32**	**0.55**	**0.18**	**0.12**	**0.17**	**0.27**	**0.31**	**0.24**	**0.08**
Null deviance	**269**	**213**	**446**	**840**	**649**	**1003**	**1023**	**1009**	**40.45**
Residuals deviance	187.31	103	372	743	500	710	735	789	**36.55**
Residuals DF	112	109	112	112	111	111	110	110	103
Family used	QB	QB	QB	QB	QB	QB	QB	QB	G
Model’s *p*-value	0.000	0.000	0.000	0.000	0.000	0.000	0.000	0.000	**0.001**
Bonferroni’s	0.000	0.000	0.000	0.001	0.000	0.000	0.000	0.000	0.010

*Independent variables included the composite scores derived from the exploratory factor analyses (hearing loss, auditory nerve, midbrain, and cognition), gender, years of education (schooling), and age. Pseudo-R-squared (R^2^) measures are shown for each regression when empty was non-significant.*

*SINRE, speech in noise right ear, correct answers; SINLE, speech in noise left ear, correct answers; DDRE, dichotic digits right ear, correct answers; DDLE, dichotic digits left ear, correct answers; SSWRE, staggered spondaic words right ear, total number of errors; SSWLE, staggered spondaic words left ear, total number of errors; FFRE, frequency pattern right ear, correct answers; FPLE, frequency pattern left ear, correct answers; GIN, gap in noise, minimum time gap detected; QB, quasi binomial; G, gamma; DF, degree of freedom.*

*p < 0.05; **p < 0.01; ***p < 0.001.

*Bold numbers are used to highlight statistically significant values.*

## Discussion

In this study, we explored the associations between several abilities of AP with cognition and physiological measures of hearing function in presbycusis without dementia. All the associations and interactions were obtained in subjects older than 65 years of age, without dementia, and with no previous use of hearing aids. Therefore, we excluded any neuroplasticity cofounding factor. Some functions, such as speech recognition in background noise, correlated preferably with hearing thresholds, while other skills such as dichotic listening were mainly associated with cognition.

### Speech Perception in Background Noise

#### Speech-in-Noise Difficulty Level

In our study, the performance variability in the SIN task correlated with hearing thresholds and age in both ears but had no interaction with cognition. These results differed from the report by Dryden et al. (2017), in which they reviewed 25 articles, identifying a significant correlation between SIN and cognition, mainly with the domain of working memory. This discrepancy could be explained by the different levels of difficulty in the SIN tasks, suggesting that cognition is a relevant factor in a challenging task, such as repeating a complete sentence, while hearing sensitivity is more relevant in a simple task, such as repeating a phoneme or word.

Our study is in agreement with other reports that used word recognition tests in elderly subjects that have also been unable to identify a correlation with cognitive domains. For instance, Humes et al. (1994) evaluated a group of fifty elderly adults using different speech recognition tests that targeted the repetition of syllables or words (Humes et al., 1994). The variance in performance was explained largely by hearing thresholds. Sheft et al. (2015) assessed a group of 124 elderly adults using a word recognition test (Sheft et al., 2015). Only age and auditory thresholds correlated with SIN. In summary, taking the present results and previous evidence, we propose that hearing sensitivity is a relevant factor in presbycusis, when SIN is assessed in a relatively easy task, such as recognition of monosyllabic words in noise.

It is important to highlight that the test we used to assess speech discrimination in background noise is the only validated test for clinical assessments in our country. It has a +10 dB SNR and was easily performed by our volunteers, which could explain possible ceiling effects in SIN performance (Figure 2). We proposed that the use of a more difficult SIN test could recruit additional cognitive resources besides hearing.

#### Hearing Loss Severity

Another important factor to consider for SIN performance is hearing loss severity. In our study, we involved adults older than 65 years of age, without previous use of hearing aids. As, in Chile, hearing aids are guaranteed for individuals with hearing loss greater than 40 dB HL, it is relatively difficult to find presbycusis patients with more than 40 dB HL of hearing loss not using hearing aids. In addition, as we followed the recommendations of the American Academy of Audiology (2010) guidelines for the evaluation of AP, we did not consider subjects with hearing loss greater than 50 dB HL. These factors led us to bias recruitment for mild to moderate hearing loss, precluding the extension of our results to presbycusis with severe hearing loss.

### Dichotic Listening

We used two tests for the evaluation of dichotic speech: dichotic digits and SSW. For dichotic digits, 18 and 12% of the variability in performance for the right and left ears correspondently was explained by cognition. These pseudo-*R*-squared values were near the cut-off value of 0.15 that we used, showing that other variables not included in our study could be important. Previous reports have described a correlation between dichotic digits and cognition. Gates evaluated a group of 313 adults aged between 71 and 96 years (Gates et al., 2010). The cognitive assessment included TMT-A, TMT-B, and working memory. A composite cognitive score, similar to the one we achieved with the EFA, explained 16% of the variance in dichotic digits (Gates et al., 2010). Fischer studied a group of 3,655 adults, aged between 21 and 100 years, with a free recall of three digits. A cognitive evaluation was done using MSSE. Five factors, including age, sex, education, hearing loss, and MMSE, accounted for 22.7% of the variance in dichotic digits (Fischer et al., 2017). In the study by Gates et al. (2010), 12% of the subjects were users of hearing aids, and neuroplasticity could be a non-controlled variable affecting the results. Nevertheless, the variance attributable to cognition had a similar value to our results. The study by Fischer et al. (2017) used a cognitively more demanding challenge (three digits), and this could explain why cognition had a higher load in the model.

Hearing and cognition were significant variables for SSW and explained 17% of the variability in the right ear and 27% of the variability in the left ear. Since SSW results were expressed as values corrected by word discrimination score, it is not surprising that in addition to cognition, hearing loss was also a significant predictor.

Even though we identified significant models for SSW and dichotic digits, these were able to explain less than 30% of the variance in the results. Other variables could be considered in the assessment of dichotic speech in elderly subjects. One of these could be interhemispheric communication. The dichotic listening paradigm makes the asymmetry between cerebral hemispheres evident (Lazard et al., 2012). With the exposure of verbal input to both ears simultaneously, the information from the right ear predominates over the left ear. Kimura described this observation in the early 60s as the right ear advantage (REA) (Kimura, 1967; Jerger and Martin, 2004). The information from the right ear ascends mainly to the contralateral cerebral hemisphere directly to the language center in the left hemisphere. Conversely, the information from the left ear ascends mainly to the right hemisphere and must cross through the corpus callosum to the language center in the left hemisphere. Information from functional and structural studies supports this theory (Hugdahl and Westerhausen, 2016). The REA persists in aged subjects (Jerger et al., 1994; Westerhausen et al., 2015; Cañete et al., 2020), and this can have a clinical relevance since, in some cases, the use of bilateral hearing aids could result in interference rather than improvement in hearing perception. Our results on dichotic digits and SSW confirmed the presence of REA (Table 2).

### Temporal Resolution

Two tests explored temporal resolution of the auditory signal: GIN detection and FP. Cognition was a significant variable in the model of FP for both ears, while hearing had no role. Similar results have been published before (Sheft et al., 2015; Murphy et al., 2018). Two additional variables emerged as significant in the models of FP: gender for the right ear and midbrain for the left ear. Gender differences in the performance of FP in adults or elderly subjects have not been reported earlier (Sanchez et al., 2008; Majak et al., 2015). Other studies do not explore gender differences (Murphy et al., 2018). Hearing levels do not account for the difference observed in our participants, since the variable “hearing” had no significance in the model. Further research is needed to clarify this issue.

The second test we used for exploring the temporal processing was GIN detection across the channel. The test was not easy to explain to our subjects, and a percentage of them were not able to execute it (8%). The model including cognition had a pseudo-*R* of 0.08 and was considered not significant. O’Brien et al. (2021) assessed a group of 213 subjects aged > 50 years and found no correlation between auditory gap detection measured by using the Adaptive Test of Temporal Resolution and cognitive domains. Even though the test we chose to explore GIN discrimination was cognitively challenging, our results did not confirm a correlation between across channel GIN detection and cognition.

### Study Limitations

Our volunteers were mostly women. An effort was made to control gender bias by including gender as a variable in our models. Inclusion criteria required normal hearing or mild to moderate presbycusis participants that could execute tests for AP, thus our results cannot be extended to severe hearing loss. We selected AP tests that were available in Spanish and previously validated in our population (Fuente and McPherson, 2006; Cañete et al., 2020). With this selection, we compromised other aspects, for example, the SIN test we used was easy for our subjects and had a ceiling effect.

## Conclusion

In a population of elderly subjects with normal hearing levels, or mild to moderate presbycusis (<40 dB HL), the comprehension of the auditory message relied differently on the hearing levels and cognition. Speech perception of monosyllabic words in background noise was associated with hearing levels, while cognition was associated with dichotic listening and FP. Importantly, these findings were not related to neuroplasticity, since none of the subjects had previous use of hearing aids.

## Data Availability Statement

The original contributions presented in the study are included in the article/supplementary material, further inquiries can be directed to the corresponding author.

## Ethics Statement

The studies involving human participants were reviewed and approved by Ethics Committee of the Clinical Hospital of the University of Chile, permission number: OAIC752/15. The patients/participants provided their written informed consent to participate in this study.

## Author Contributions

PD and CD: conceptualization and funding acquisition. MT, AL, CB, and BM: data curation. MT, FM-G, CB, and SS: formal analysis. MT, RV, FM-G, CB, AL, SS, BM, CD, and PD: investigation. FM-G and RV: methodology. PD: project administration. BM, FM-G, and RV: software. CD, MT, and PD: supervision. MT, RV, FM-G, CD, and PD: visualization. MT: writing – original draft. MT, RV, FM-G, CB, AL, SS, BM, CD, and PD: writing – review and editing, contributed to the article, and approved the submitted version.

## Conflict of Interest

The authors declare that the research was conducted in the absence of any commercial or financial relationships that could be construed as a potential conflict of interest.

## Publisher’s Note

All claims expressed in this article are solely those of the authors and do not necessarily represent those of their affiliated organizations, or those of the publisher, the editors and the reviewers. Any product that may be evaluated in this article, or claim that may be made by its manufacturer, is not guaranteed or endorsed by the publisher.
